# Unified Expression of the Quasi-Static Electromagnetic Field: Demonstration With MEG and EEG Signals

**DOI:** 10.1109/TBME.2020.3009053

**Published:** 2021-02-18

**Authors:** Samu Taulu, Eric Taulu

**Affiliations:** Department of Physics and the Institute for Learning and Brain Sciences, University of Washington, Seattle, WA 98195 USA; Institute for Learning and Brain Sciences, University of Washington

**Keywords:** Magnetoencephalography, electroencephalography, quasi-static approximation, vector spherical harmonics, non-invasive brain imaging

## Abstract

**Objective:**

Electromagnetic recordings are useful for non-invasive measurement of human brain activity. They typically sample electric potentials on the scalp or the magnetic field outside the head using electroencephalography (EEG) or magnetoencephalography (MEG), respectively. EEG and MEG are not, however, symmetric counterparts: EEG samples a scalar field via a line integral over the electric field between two points, while MEG samples projections of a vector-valued field by small sensors. Here we present a unified mathematical formalism for electromagnetic measurements, leading to useful interpretations and signal processing methods for EEG and MEG.

**Methods:**

We represent electric and magnetic fields as solutions of Laplace’s equation under the quasi-static approximation, each field representable as an expansion of the same vector spherical harmonics (VSH) but differently weighted by electro- and magnetostatic multipole moments, respectively.

**Results:**

We observe that the electric and the magnetic fields are mathematically symmetric but couple to the underlying electric source distribution in distinct ways via their corresponding multipole moments, which have concise mathematical forms. The VSH model also allows us to construct linear bases for MEG and EEG for signal processing and analysis, including interference suppression methods and system calibration.

**Conclusion:**

The VSH model is a powerful and simple approach for modeling quasi-static electromagnetic fields.

**Significance:**

Our formalism provides a unified framework for interpreting resolution questions, and paves the way for new processing and analysis methods.

## I. Introduction

**E**LECTRIC properties of objects can be studied noninvasively by measuring relevant aspects of the associated electromagnetic (EM) field and by analyzing the recorded data with the aid of mathematical algorithms. Of particular interest to us are applications in neuroimaging where such non-invasive measurements facilitate interpretation of the dynamic brain functions while patients and subjects remain safe and comfortable. Related applications also arise in other scientific domains, e.g., in the study of geomagnetism.

In neuroimaging, the most commonly applied EM-based measurement modalities are electroencephalography (EEG) and magnetoencephalography (MEG). The former is the recording of the electric potential differences on the scalp and the latter is the measurement of the magnetic field outside the head. Both EEG and MEG employ sensors in the form of a multi-channel array, providing a spatially discretized representation of the underlying field distribution. In MEG, in particular, the optimal spatial sampling of the field, in terms of capturing all relevant information, has been studied in detail [1],[2]. The EEG and MEG signals are also sampled in time by the data acquisition systems and the resulting data can be represented as spatiotemporal data matrices comprised of channels along one dimension and time points along the other. Spatial and temporal sampling should be sufficiently dense to avoid loss of detectable high-frequency components both in the spatial and temporal domains.

One of the most fundamental tasks in the interpretation of multi-channel EM signals is finding tractable mathematical expressions that connect the electric activity in the source space to the EM field in the sensor space. These expressions can be applied in inverse modeling, which is the task of reconstructing a plausible electric source distribution (e.g., in the brain) given the measured data matrix. In EEG and MEG, the mathematical models are simplified to some extent due to the fact that we can apply the quasi-static approximation of Maxwell’ sequations [3]. Pioneering work on the EEG and MEG models was published in the 1960s and 1970s (e.g., [4], [5], [6]) in which mathematical expressions were derived for the EEG and MEG signals given a distribution of electric sources related to activity in the brain or in the heart. The resulting forward models apply to different levels of complexity of the electric conductivity profile, which determines the accuracy of the modeling of the passive volume currents that arise from the primary currents in order to complete the current loops in the conducting tissue [3]. The primary currents are the physiologically interesting currents that typically arise in conjunction of the so-called post-synaptic potentials in the brain.

The electric and magnetic fields are generated by the same source distribution, with the latter component being solely related to the movement of the electric charge, i.e., the magnetic field is generated by electric currents. However, mainly due to the differences in the significance of the volume current contributions, EEG and MEG signals are often considered to be quite different in terms of feasibility of reconstructing accurate source distributions via inverse modeling. This is due to the fact that the magnetic field appears to be insensitive to radially oriented currents, and, on the other hand, volume current contributions can be represented as current elements normally oriented to surfaces where electric conductivity changes. As the conductivity gradient surfaces in the head, related to, e.g., skull and scalp, are close to being spherically symmetric, the volume current contributions are close to radial and one can conclude that the majority of the MEG signal contribution arises from tangentially oriented primary currents [3]. Thus, the MEG signals are easier to model because a high-precision representation of the conductivity profile is not as crucial as it is for EEG. In this framework, a potential trade off of the facility of modeling MEG signals is that MEG is not as sensitive to the parts of the cortex with radially oriented pyramidal neurons. This would mean that MEG efficiently only sees about 2/3 of the cortex while EEG is, in principle, sensitive to all areas of the cortex [7]. On the other hand, EEG measurements are argued to experience more widespread spatial topographies due to the effect of electric inhomogeneities [7], which would indicate difficulties in distinguishing between sources located close to each other in the brain, although the importance of relatively dense sampling with more than 100 EEG electrodes has also been emphasized (see, e.g. [8]).

Non-invasive study of the human brain based on electromagnetism started with early EEG recordings [9] and several decades later the first MEG measurements were conducted [10]. A fundamental mismatch between these measurement modalities is that EEG measures a scalar field—the electric potential difference distribution—while MEG measures the absolute distribution of a quantity proportional to the vector-valued density of the magnetic flux. The electric potential difference between two points, on the other hand, can be represented as a quantity proportional to the work that the vector-valued electric field does along a path between those points. Even though in the static or quasi-static formulation the electric field can be uniquely derived as the gradient of the potential distribution, it is possible that a direct measurement of the electric field could yield more information about the underlying source distribution than the measurement of the electric potential difference. Furthermore, the brain-induced electric and magnetic fields can be analyzed as a symmetric pair of vector fields as will be shown in this article.

Here, we unify the mathematical expressions arising from the quasi-static Maxwell’s equation, which allow us to represent the full quasi-static EM field outside the volume containing the sources (electric charge or current) as a combination of vector-valued basis functions that are common to both the electric and the magnetic field. Then we show how the amplitude coefficients of these basis functions are related to the underlying source distribution in a way that makes the electric and magnetic fields distinct from one another. We then develop a linear basis model for the multi-channel EM recordings that allows us to predict the essential number of degrees of freedom of these measurements. Useful applications readily follow, such as sensor calibration and algorithmic interference suppression. While MEG and EEG measurements are the practical, existing modalities that are covered by our theoretical framework, our letter predicts potential benefits of measuring the brain-related electric field, rather than the electric potential, if such measurements become feasible.

## II. Methods

### A. Electromagnetic Field Under the Quasi-Static Approximation

A complete description of the electric and magnetic fields is given by Maxwell’s equations:

(1) $$\nabla \cdot E=\frac{\rho }{\epsilon_{0}},$$

(2) $$\nabla \times E=-\frac{\partial B}{\partial t},$$

(3) ∇⋅B=0,

and

(4) $$\nabla \times B=\mu_{0}\left(J+\epsilon_{0}\frac{\partial E}{\partial t}\right).$$

In these equations, *ρ* indicates charge density, *ε*_0_ is the permittivity of vacuum, *μ*_0_ is the permeability of vacuum, **J** indicates current density, and $\mu_{0}\epsilon_{0}\partial E/\partial t$ is commonly known as the displacement current (see, e.g., [11]), which means that a time-dependent electric field acts similarly to electric current as a source of the magnetic field.

Next we will investigate specific spatiotemporal properties of the electric and magnetic fields from the point of view of MEG and EEG measurements. First of all, we can ignore any time retardation effects due to the very short distance between the measurement sensors and the brain. Next, we investigate the relation between the physical dimensions of our measurement and the electromagnetic wavelength associated with the dominant time scale of the object of interest. If these physical dimensions are smaller, then the quasi-static approximation is justified (see, e.g., [11], [12]). More specifically, quasi-static systems can be divided into subgroups [12] of which the electro-quasi-static and magneto-quasi-static ones are the most relevant to us. In the former subgroup, the dynamic electric and magnetic fields can be expressed by the otherwise static Coulomb and Biot-Savart laws, respectively. The magneto-quasi-static system additionally assumes the static continuity equation ∇⋅J=0 but allows inductive effects to act as a source of the electric field. The quasi-static approximation, as it is defined in MEG and EEG, is a hybrid of the above description in the sense that it ignores the time derivatives of both the electric and the magnetic fields, and also assumes that ∇⋅J=0. A detailed justification of this approximation can be found, e.g., in [3] and [5]. Briefly, ∂E/∂t can be ignored due to the fact that considering the conductivity of the brain and the typical frequency range of brain signals, it is straightforward to show that $\left|\epsilon_{0}\partial E/\partial t\right|<<|\sigma E|$ [3]. This relation is important as J=σE acts as a source of the magnetic field and the contribution of the displacement current is thus negligible in comparison. On the other hand, again considering the typical conductivity and frequency band of MEG and EEG, the contribution of ∂B/∂t would only be significant within a length scale of more than tens of meters, which is much larger than the diameter of the head [3]. With this information, we will now treat the momentary MEG and EEG signals without reference to any time derivatives and in the subsequent parts of our article “quasi-static” refers to the condition ∂E/∂t=0 and ∂B/∂t=0. However, to emphasize the fact that the electric and magnetic fields of interest are dynamic phenomena, we introduce the time variable in the subsequent equations. The dynamics, however, together with the above consideration on the frequency band and characteristic dimensions typical of MEG and EEG warrant the application of mathematical formalism that is similar to static electromagnetism. Now, under the quasi-static approximation, we get

(5) ∇×E(r,t)=0,

where **r** is the three dimensional location where the field is evaluated and *t* indicates time. When evaluating the magnetic field in a volume free of electric currents (**J** = **0**),

(6) ∇×B(r,t)=0.

Consequently, since ∇×(∇f)=0 for any scalar field *f*, we can express both **E**(**r**, *t*) and **B**(**r**, *t*) as a gradient of a harmonic scalar potential under the quasi-static approximation. As both fields have this fundamental mathematical property, let us now in place of **E**(**r**, *t*) and **B**(**r**, *t*) introduce a generic vector field **F**(**r**, *t*) for which ∇×F(r,t)=0, and thus we can choose

(7) $$F(r,t)=-\nabla V_{\text{H}}(r,t)$$

where $V_{\text{H}}(\text{r},t)$ is a harmonic scalar potential and the term “harmonic” is due to the fact that now ∇⋅E(r,t)=0 (in a source-free volume) and ∇⋅B(r,t)=0 (since there are no magnetic monopoles), which leads to Laplace’s equation

(8) $$\nabla^{2}V_{\text{H}}(r,t)=0$$

that has the following series-form solution:

(9) $$V_{\text{H}}(r,t)=\sum_{l=0}^{\infty }\sum_{m=-l}^{l}a_{lm}(t)\frac{Y_{lm}(\theta ,\varphi )}{r^{l+1}}\;\;+\sum_{l=0}^{\infty }\sum_{m=-l}^{l}b_{lm}(t)r^{l}Y_{lm}(\theta ,\varphi ),$$

where **r** is the location (*r, θ, ϕ*) in the spherical coordinate system, at which *V*_H_ is evaluated, *Y*_*l,m*_ is the spherical harmonic function of order {*l,m*}, and $a_{lm}(t)$ and $b_{lm}(t)$ correspond to the so-called multipole moments of the two different expansions. It is important to recognize that by the source-free volume we are referring to an area outside of the head where the vector field **F**(**r**, *t*) is evaluated. Inside the head we would obviously have ρ≠0 and J≠0 which means that (9) only applies for fields outside the volume that contains electric charge or electric current. As will be shown later, the distribution of the total charge and the total current affects the $a_{lm}(t)$ multipoles and thereby affects the fields to be evaluated outside. On the other hand, the total current is commonly modeled as a superposition of primary current related to brain activity and the associated passive volume current, which is shaped by the conductivity profile of the head.

**Fig. 1.**
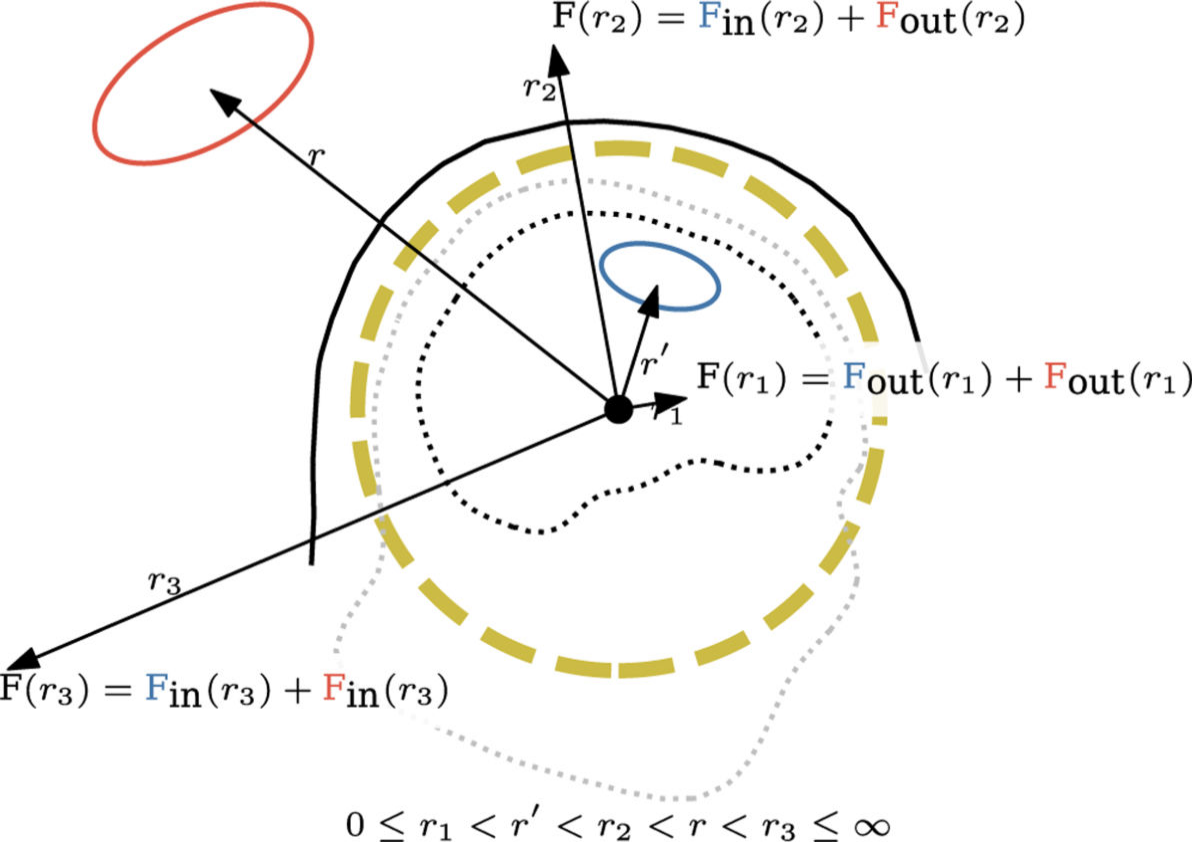
Representation of the EM fields produced by sources located in different half-spaces separated by a hypothetical spherical boundary. Two source distributions are illustrated: The blue loop that is inside the hypothetical spherical surface denoted by the thick dashed line and the red loop that is outside. For each of the illustrated field point distances (*r*_1_, *r*_2_, *r*_3_), the corresponding mathematical form is chosen for both of the two sources. For sources whose distance from the origin is larger than the distance between the field point and the origin, we choose the expansion that converges at the origin. For sources whose distance from the origin is shorter than the distance between the field point and the origin, we choose the expansion that converges at infinity. The convergence properties of the two expansions are evident from (16) and (17).

Equation (9) and its gradient represent, under the quasi-static approximation, the complete solution of the electromagnetic field outside of the source volume irrespective of any particular source configuration or electric conductivity profile. It should be noted that the term “spherical” is not related to the spherically symmetric conductor model approximation that is commonly used in MEG. Also, the term “multipole moment” represents the amplitude coefficient of a given spherical harmonic basis function with respect to a given origin of the coordinate system and is not restricted to the representation of any specific source distribution in the brain. Thus, it should not be confused with source models, such as the expansion of neural current beyond the current dipole in specific brain regions, which was proposed as a source analysis method, e.g., in [13]. The reason why we have two expansions is that the harmonic potential has to converge to zero either at *r* = 0 or at *r* = ∞ depending on whether the source generating the potential is farther from or closer to origin than **r** is, respectively. If the source is located at **r** and we evaluate the potential at **r** with the relation $r=∥r∥>\left\|r^{\prime }\right\|=r^{\prime }$, then the potential has to converge at infinity because we could take **r** arbitrarily far from the origin. Conversely, if $r^{\prime }>r$, then the potential has to converge in the origin because the source could be located at infinity. Note that in neuroimaging, the sensors recording either the electric or magnetic interaction can in principle be positioned in such a way that the signal of interest comes from the inner volume while external interference resides in the outer volume. Fig. 1 illustrates this principle.

The transition from the scalar field to the vector field **F**(**r**, *t*) is given by

(10) $$\nabla V_{\text{H}}(r,t)=\sum_{l=0}^{\infty }\sum_{m=-l}^{l}a_{lm}(t)\nabla \left(\frac{Y_{lm}(\theta ,\varphi )}{r^{l+1}}\right)\;\;+\sum_{l=0}^{\infty }\sum_{m=-l}^{l}b_{lm}(t)\nabla \left(r^{l}Y_{lm}(\theta ,\varphi )\right),$$

where, according to [14], [15],

(11) $$\nabla \left(\frac{Y_{lm}}{r^{l+1}}\right)=\frac{1}{r^{l+2}}\left[-(l+1)Y_{lm}e_{\text{r}}+\frac{\partial Y_{lm}}{\partial \theta }e_{\theta }+\frac{\text{i}mY_{lm}}{\sin\theta }e_{\varphi }\right]$$

and

(12) $$\nabla \left(r^{l}Y_{lm}\right)=r^{l-1}\left(lY_{lm}e_{\text{r}}+\frac{\partial Y_{lm}}{\partial \theta }e_{\theta }+\frac{\text{i}mY_{lm}}{\sin\theta }e_{\varphi }\right).$$

Here **e**_r_, **e**_*θ*_ and **e**_*ϕ*_ are the orthogonal unit vectors in the spherical coordinate system. Now let us define the following vector spherical harmonic (VSH) functions:

(13) $$\nu_{lm}(\theta ,\varphi )=-(l+1)Y_{lm}e_{\text{r}}+\frac{\partial Y_{lm}}{\partial \theta }e_{\theta }+\frac{\text{i}mY_{lm}}{\sin\theta }e_{\varphi }$$

(14) $$\omega_{lm}(\theta ,\varphi )=lY_{lm}e_{\text{r}}+\frac{\partial Y_{lm}}{\partial \theta }e_{\theta }+\frac{\text{i}mY_{lm}}{\sin\theta }e_{\varphi }.$$

Then, based on (7) and (10), we have the following generic expression that satisfies the physical properties of a quasi-static electromagnetic field:

(15) $$F(\text{r},t)=-\sum_{l=0}^{\infty }\sum_{m=-l}^{l}a_{lm}(t)\frac{\nu_{lm}(\theta ,\varphi )}{r^{l+2}}\;\;-\sum_{l=0}^{\infty }\sum_{m=-l}^{l}b_{lm}(t)r^{l-1}\omega_{lm}(\theta ,\varphi ).$$

As we shall see later, there is a specific relationship between the electric source (charge or current) distribution and the multipole moments that determines whether **F**(**r**, *t*) represents the electric or magnetic field. The theory of the multipole expansions in the case of time-varying fields has been investigated in detail, e.g., in [16], but under the quasistatic-approximation adopted in MEG and EEG, we can apply the electro- and magneto-static multipole moments. This means that for each moment in time, we can exploit the static expressions.

Specifically, for the geometry of neuroimaging-specific measurements, we can separate the contributions corresponding to sources in the “inner” and “outer” volumes as follows:

(16) $$F_{\text{in}}(r,t)=-\sum_{l=0}^{\infty }\sum_{m=-l}^{l}a_{lm}(t)\frac{\nu_{lm}(\theta ,\varphi )}{r^{l+2}}$$

and

(17) $$F_{\text{out}}(r,t)=-\sum_{l=0}^{\infty }\sum_{m=-l}^{l}b_{lm}(t)r^{l-1}\omega_{lm}(\theta ,\varphi ).$$

Then, the field outside the head is a linear superposition of the contribution of the inner (mostly brain) and outer (interference) sources:

(18) $$F(r,t)=F_{\text{in}}(r,t)+F_{\text{out}}(r,t).$$

### B. Difference Between the Electric and Magnetic Fields

As shown in the previous section, the mathematical form of the generic vector field **F**(**r**, *t*) in (15) is satisfactory for both **E**(**r**, *t*) and **B**(**r**, *t*) under the quasi-static approximation. Furthermore, the VSH functions combined with the corresponding radial parts ($1/r^{l+2}$ or $r^{l-1}$) that **F**(**r**, *t*) is composed of are simply mathematical properties of a particular point in space at which the field is evaluated. Consequently, the only way how **E**(**r**, *t*) and **B**(**r**, *t*) can differ beyond being scaled by their corresponding overall scales $1/\epsilon_{0}$ and $\mu_{0}$, respectively, is by having different multipole moments, i.e., the electric and magnetic fields produced by sources in the inner volume take the forms

(19) $$E(r,t)=c_{\text{e}}\sum_{l=0}^{\infty }\sum_{m=-l}^{l}a_{lm}^{\text{e}}(t)\frac{\nu_{lm}(\theta ,\varphi )}{r^{l+2}}$$

and

(20) $$B(\text{r},t)=c_{\text{b}}\sum_{l=0}^{\infty }\sum_{m=-l}^{l}a_{lm}^{\text{b}}(t)\frac{\nu_{lm}(\theta ,\varphi )}{r^{l+2}},$$

where $a_{lm}^{\text{e}}$ and $a_{lm}^{\text{b}}$ are the electrostatic and magnetostatic multipole moments, respectively, and the essential difference between the electric and the magnetic fields is now restricted to the difference between $a_{lm}^{\text{e}}$ and $a_{lm}^{\text{b}}$. The overall scaling coefficients are

(21) $$c_{\text{e}}=-\frac{1}{\epsilon_{0}}$$

and

(22) $$c_{\text{b}}=-\mu_{0},$$

where we have inserted the minus sign for convenience as it appears as the overall sign of expression in (15).

Bronzan [17] provided a concise mathematical formalism of the electrostatic and magnetostatic series expansions corresponding to the following multipole moments, respectively:

(23) $$a_{lm}^{\text{e}}(t)=\frac{1}{2l+1}\int_{v^{\prime }}r^{\prime l}Y_{lm}^{*}\left({\text{Ω}}^{\prime }\right)\rho \left(r{,}^{\prime }t\right)dv^{\prime }$$

and

(24) $$a_{lm}^{\text{b}}(t)=\frac{-1}{\left(2l+1)(l+1\right)}\int_{v^{\prime }}r^{\prime l}Y_{lm}^{*}\left({\text{Ω}}^{\prime }\right)\nabla^{\prime }\cdot \left[r^{\prime }\times J\left(r{,}^{\prime }t\right)\right]dv^{\prime }.$$

Here, the primed coordinates correspond to a volume containing the electric source distribution, *v*′ indicates volume integral over the whole source volume, and the field points **r** are located outside of it. Also, we have simplified the expressions by denoting ${\text{Ω}}^{\prime }\equiv \left(\theta {,}^{\prime }\varphi^{\prime }\right)$. Equation (24) has been simplified in [15] to read

(25) $$a_{lm}^{\text{b}}(t)=\frac{\text{i}}{2l+1}\sqrt{\frac{l}{l+1}}\int_{v^{\prime }}r^{\prime l}X_{lm}^{*}\left({\text{Ω}}^{\prime }\right)\cdot J\left(r{,}^{\prime }t\right)dv^{\prime },$$

where i is the imaginary unit and $X_{lm}\left({\text{Ω}}^{\prime }\right)$ is a tangential VSH function defined in [14], [15]:

(26) $$X_{lm}(\text{Ω})=\frac{-mY_{lm}(\text{Ω})}{\sqrt{l(l+1)}\sin\theta }e_{\theta }+\frac{-i}{\sqrt{l(l+1)}}\frac{\partial Y_{lm}(\theta )}{\partial \theta }e_{\varphi }.$$

So, the physical difference between **E**(**r**,*t*) and **B**(**r**,*t*) is restricted to the integration kernels:

(27) $$k_{lm}^{\text{e}}\left(r{,}^{\prime }t\right)=Y_{lm}^{*}\left({\text{Ω}}^{\prime }\right)\rho \left(r{,}^{\prime }t\right)$$

and

(28) $$k_{lm}^{\text{b}}\left(r{,}^{\prime }t\right)=X_{lm}^{*}\left({\text{Ω}}^{\prime }\right)\cdot J\left(r{,}^{\prime }t\right),$$

which leads to the following expressions for the electrostatic and magnetostatic multipole moments, respectively:

(29) $$a_{lm}^{\text{e}}(t)=\frac{1}{2l+1}\int_{v^{\prime }}r^{\prime l}k_{lm}^{\text{e}}\left(r{,}^{\prime }t\right)dv^{\prime }$$

(30) $$a_{lm}^{\text{b}}(t)=\frac{\text{i}}{2l+1}\sqrt{\frac{l}{l+1}}\int_{v^{\prime }}r^{\prime l}k_{lm}^{\text{b}}\left(r{,}^{\prime }t\right)dv^{\prime }.$$

Note that if we were to observe the time derivative ∂E(r,t)/∂t instead of **E**(**r**,*t*), the integration kernel for ∂E(r,t)/∂t would be directly related to the underlying current, as can be readily seen from (19) and (23):

(31) $$\frac{\partial k_{lm}^{\text{e}}\left(r{,}^{\prime }t\right)}{\partial t}=-Y_{lm}^{*}\left({\text{Ω}}^{\prime }\right)\nabla \cdot J\left(r{,}^{\prime }t\right),$$

where we have utilized the continuity equation $\partial \rho \left(r{,}^{\prime }t\right)/\partial t=-\nabla \cdot J\left(r{,}^{\prime }t\right)$.

Note that the *l* = 0 term in (19) and (20) corresponds to electric and magnetic monopoles, respectively. While in the case of the electric fields this corresponds to point-like electric charge, magnetic monopoles do not exist according to present theories. This is consistent with the fact that $X_{0,0}=0$ (see [14]), which, according to 25, leads to $a_{0,0}^{\text{b}}=0$, i.e., the term *l* = 0 has no contribution to **B**(**r**,*t*).

### C. Sensitivity of the Electric and Magnetic Fields to the Geometry of the Underlying Source Distribution

Now we have reduced the difference between **E**(**r**, *t*) and **B**(**r**, *t*) to the difference between the source-space kernels $k_{lm}^{\text{e}}\left(r{,}^{\prime }t\right)$ and $k_{lm}^{\text{b}}\left(r{,}^{\prime }t\right)$ that connect the electric charge and current distributions to the corresponding electric and magnetic fields. Based on (27), (28), and (31), we immediately observe two geometrical principles:

1. The electric field is in principle sensitive to any electric charge or electric current in the source volume.
2. The magnetic field is only sensitive to a tangential projection of the electric current. It should be noted, however, that in this context the tangential and radial directions depend on the choice of the origin of the coordinate system. Thus, we cannot conclude that the magnetic field is physically insensitive to radial current segments with respect to any absolute frame of reference.

An interesting consequence of the second of these two principles is that with respect to a chosen origin, any radially oriented currents any where in the brain will correspond to $k_{lm}^{\text{b}}\left(r{,}^{\prime }t\right)=0$, i.e., they will not contribute to the magnetic field. This is important because the volume currents in the brain can be expressed with equivalent elementary current elements at conductivity gradient boundaries, oriented normally to the boundary surface [18], and in the head geometry these boundaries are close to being spherically symmetric. In perfect spherical symmetry, with the origin set in the center of the spherical conductor, volume currents would appear to have no effect on the magnetic field and all contribution would come from tangential portions of primary currents. In fact, Ilmoniemi derived the forward field of an equivalent tangential primary current dipole by showing that the dipole and the associated volume currents can be expressed as a current triangle whose magnetic field is not affected by the radially oriented volume current contributions [19], [20]. The forward field was later simplified by Sarvas [21]. However, if the origin of the coordinate system is moved away from the center, the volume currents do have to be explicitly modeled as the volume current elements on the spherical boundaries now correspond to $k_{lm}^{\text{b}}\left(r{,}^{\prime }t\right)\neq 0$.

Based on (27), (28), and (31), the electric field, as compared to the magnetic field, gets contribution from a larger distribution of the underlying sources. Generally, for any location r’ at which $\rho \left(r^{\prime },t\right)\neq 0$ or $\nabla \cdot J\left(r^{\prime },t\right)\neq 0$, we have $k_{lm}^{\text{e}}\left(r{,}^{\prime }t\right)\neq 0$ or $\partial k_{lm}^{\text{e}}\left(r{,}^{\prime }t\right)/\partial t\neq 0$, respectively. Thus, in the case of the electric field, the choice of the origin does not create any apparent “silent” sources unlike in the case of the magnetic field where tangential portions of the current loops always appear to produce a zero signal, but the definition of tangential direction depends on the origin.

So far, we have developed our mathematical theory for the E(r*,t*) and B(r*,t*) fields and shown that they are composed of the same VSH functions that are weighted differently by the associated multipole moments. However, EEG measures the distribution of the electric potential on the head surface, which corresponds to spatial sampling of the scalar field *V* (**r**, *t*) from which the electric field can be derived as E(r,t)=−∇V(r,t). Based on (9), outside the source volume the expression for the electric potential produced by sources in the inner volume of Fig. 1 is

(32) $$V(\text{r},t)=c_{\text{e}}\sum_{l=0}^{\infty }\sum_{m=-l}^{l}a_{lm}^{\text{e}}(t)\frac{Y_{lm}(\theta ,\varphi )}{r^{l+1}}.$$

Consequently, the component of the electric potential for which ∇⋅E(r,t)=0 holds (otherwise *V* would have to be derived from Poisson’s equation) is composed of scalar spherical harmonic functions weighted by the same electrostatic multipole moments as the VSH functions of the electric field. Thus, one could conclude that a measurement of the electric potential or the electric field would contain equal information. We will, however, show in Section III-A that some of the information contained in the electric field could be attenuated in the electric potential due to the diminishing of spatially complex features.

## III. Some Implications of the Unified VSH Formalism

### A. Signal Space Representation and Dimensionality of Measured Data

MEG and EEG measurements consist of taking spatiotemporal samples of the magnetic field or electric potential distribution. For completeness, we will also consider the case of taking similar samples of the electric field even though such instrumentation does not currently exist for the purposes of neuroimaging. The sampling of these fields is done by several sensors that are positioned on or around the subject’s head in a manner that provides an informative distribution of values that are proportional to the underlying field. The recorded values are sampled in time and the resulting data is in the form a matrix that contains a discretized representation of the spatial and temporal features of the electromagnetic signals. We define the data matrix of a measurement system containing *N* sensors as follows:

(33) $$D=\left[\text{d}\left(t_{1}\right)d\left(t_{2}\right)\ldots \text{d}\left(t_{m}\right)\right],$$

where **d**(*t*_*j*_) is the *N*-dimensional signal vector measured at time instant *t*_*j*_, i.e., the data matrix D has dimensions of *N* × *m*. Let us now investigate any momentary signal vector d and attempt to construct a general signal space for it, i.e., express it as a linear combination of basis vectors in the same manner as a stationary temporal signal can be expressed as a linear combination of sine and cosine terms. Based on (19) and (20), we know that a sensor located at r_*j*_ will produce an output signal that is proportional to a superposition of terms

(34) $$s_{lm,j}=f_{j}\left(\frac{\nu_{lm}\left({\text{Ω}}_{j}\right)}{r_{j}^{l+2}}\right),$$

where *f*_*j*_ is some function that transforms the value of the field into a measurable quantity. For example, in an MEG measurement this function typically corresponds to taking the flux of the magnetic field component through the area of the corresponding pick-up loop [20]. Now, we can define a basis vector **s**_*lm*_ that consists of the values of (34), i.e., $s_{lm}={\left[s_{lm,1}s_{lm,2}\ldots s_{lm,N}\right]}^{\text{T}}$. Thus, when discretizing the vector field, we can represent the *N*-channel momentary measurement vector as

(35) $$d(t)=\sum_{l=0}^{\infty }\sum_{m=-l}^{l}a_{lm}(t)s_{lm}.$$

This vector representation applies both to the measurement of the electric and the magnetic field. It is physically meaningful when we think of the amplitude coefficients as $a_{lm}^{e}$ for the former and $a_{lm}^{b}$ for the latter field. Note that in the determination of the **s**_*lm*_ vectors we have to choose an origin for the VSH expansion and this origin should be placed in the brain area in such a way that the distance relations shown in Fig. 1 apply. Otherwise, the representation of the brain signals would be biased. For example, if the origin were placed to the area of the left hemisphere of the cortex, then the signals arising from the right hemisphere would not be representable by our VSH model. Therefore, the results shown in the subsequent sections depend on proper placement of the origin.

Equation (35) is helpful in constructing a full physics-based signal basis for the multichannel measurements in case we can truncate the expansion at an order that results in a number of expansion terms that is smaller than the number of measurement sensors. In order to investigate this analytically, we express (19) and (20) with the help of (29) and (30), which leads to

(36) $$E(r,t)=\frac{c_{\text{e}}}{r^{2}}\sum_{l=0}^{\infty }\left(\frac{1}{2l+1}\right)\sum_{m=-l}^{l}\nu_{lm}(\text{Ω})\int_{v^{\prime }}{\left(\frac{r^{\prime }}{r}\right)}^{l}k_{lm}^{e}\left(r{,}^{\prime }t\right)dv^{\prime }$$

and

(37) $$B(r,t)=\frac{c_{\text{b}}}{r^{2}}\sum_{l=0}^{\infty }\left(\frac{i}{2l+1}\sqrt{\frac{l}{l+1}}\right)\;\;\times \sum_{m=-l}^{l}\nu_{lm}(\text{Ω})\int_{v^{\prime }}{\left(\frac{r^{\prime }}{r}\right)}^{l}k_{lm}^{b}\left(r{,}^{\prime }t\right)dv^{\prime }.$$

Similarly, for the electric potential we have

(38) $$V(r,t)=\frac{c_{\text{e}}}{r}\sum_{l=0}^{\infty }\left(\frac{1}{2l+1}\right)\sum_{m=-l}^{l}Y_{lm}(\text{Ω})\int_{v^{\prime }}{\left(\frac{r^{\prime }}{r}\right)}^{l}k_{lm}^{e}\left(r{,}^{\prime }t\right)dv^{\prime }.$$

These equations indicate that the expansions have a hierarchical structure in which the higher orders of *l*, corresponding to higher spatial complexity, decay as a function of distance faster than the lower orders due to the ${\left(r^{\prime }/r\right)}^{l}$ term. Thus, the farther the measurement point is from the source, the smoother the spatial structure of the field is, indicating that we can truncate the expansion at some finite order *L* and only retain the components corresponding to *l* ≤ *L* without losing significant information. In other words, distance from the source acts as aspatial low-pass filter that smooths the spatial topography of the measured signal regardless of the underlying conductivity structure. Let us then write (35) in the truncated form

(39) $$\text{d}(t)\approx \sum_{l=0}^{L}\sum_{m=-l}^{l}a_{lm}(t)s_{lm}\equiv Sx(t),$$

where *L* is the truncation order and the *N* × *n*-dimensional basis matrix S contains the corresponding **s**_*lm*_ vectors and the *n*-dimensional vector **x**(*t*) contains the associated multipole moments *a*_*lm*_(*t*). Using the Moore-Penrose pseudoinverse (^†^) of S, the *a*_*lm*_(*t*) terms in practice are estimated to simply be the elements of the vector **S**^†^**d**(*t*). Since each order of *l* contains 2*l* + 1 multipole moments, the dimension of the basis is

(40) $$n={\left(L+1\right)}^{2}.$$

Note that in the case of the magnetic field, we exclude the *l* = 0 component, leading to $n={\left(L+1\right)}^{2}-1$. It has been shown in the case of MEG that a suitable truncation order is *L* = 8, resulting in *n* = 80 dimensions or degrees of freedom that cover all detectable signals for any realistic neural current distribution [15]. Due to the similarity of the magnetic and electric fields apart from the values of the multipole moments, we expect the electric field to correspond to similar dimensionality that was found in the case of MEG.

Next, we will experimentally study the truncation order for traditional EEG measurements that are based on the voltage distribution. The signal basis of (39) applies in EEG as well, but instead of (34), the elements of the signal vector are expressed as

(41) $$s_{lm,j}^{\text{EEG}}=\frac{Y_{lm}\left(\theta_{j},\varphi_{j}\right)}{r_{j}^{l+1}},$$

where the role of the reference electrode can be taken into account by subtracting its $Y_{lm}/r^{l+1}$ component in (41). In principle, there could be sources of electric field in the immediate vicinity of the EEG electrodes, which are attached to the head surface, but the following experimental results indicate that a relatively low-rank linear basis described above can be a rather accurate model for realistic EEG signals.

**Fig. 2.**
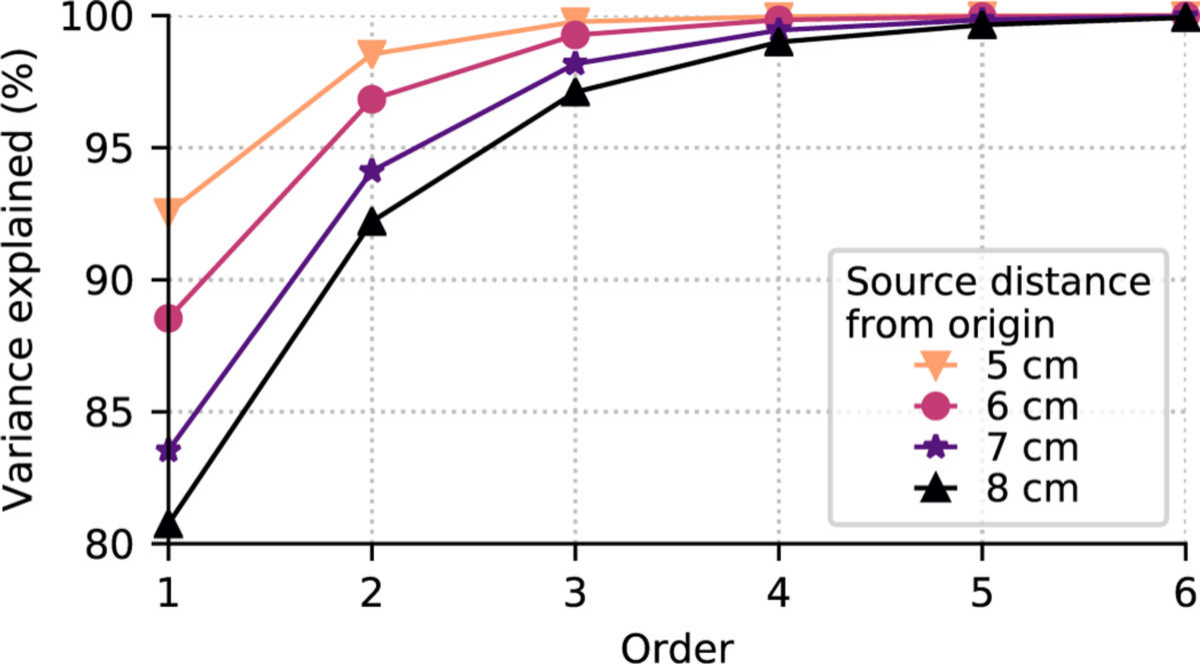
The variance explained converges rapidly as a function of expansion order. A forward model was computed for free orientation sources (i.e., three orthogonally oriented dipoles at location) and a spherical conductor model on a volumetric grid with 7 mm spacing within the sphere. The variance of the forward model explained as a function of expansion order (abscissa) and source distance (lines) is shown. Each curve was computed using all dipoles within 0.5 cm of the given distance from the sphere center (5, 6, 7, or 8 cm).

Fig. 2 shows the percentage of signal energy explained by the expansion of (39) as a function of truncation order *L*_EEG_. A forward model was computed using a spherical conductor model and 60 realistic EEG channel locations (from the public MNE-Python sample dataset [22], [23]) for a volumetric grid (7 mm spacing) of current dipoles. All computations were performed using MNE-Python unless otherwise noted. The spherical conductor model used the default values from MNE-Python: radius estimated from digitization as 91 mm, with four shells (parentheticals: relative radii in normalized units; conductivity in S/m) representing the brain (0.90; 0.33), CSF (0.92; 1.0), skull (0.97; 0.004), and scalp (1.0; 0.33). The variance of the forward model **G** explained by the VSH expansion at the origin determined by a sphere fit to digitized head points ([−4.1,16.0,51.7] mm in the Neuromag head coordinate frame) was then computed as a function of expansion order and source radius (to the nearest centimeter) for each source by projecting the forward to multipole moments and back, and computing the ratio of the matrix norms as:

$$100\times \frac{\left\|SS^{†}G\right\|}{∥G∥}.$$

This can be thought of as equivalent to the variance explained by the expansion of the time courses of independent, identically distributed (IID) neural white noise sources placed at each location in the volumetric grid.

**Fig. 3.**
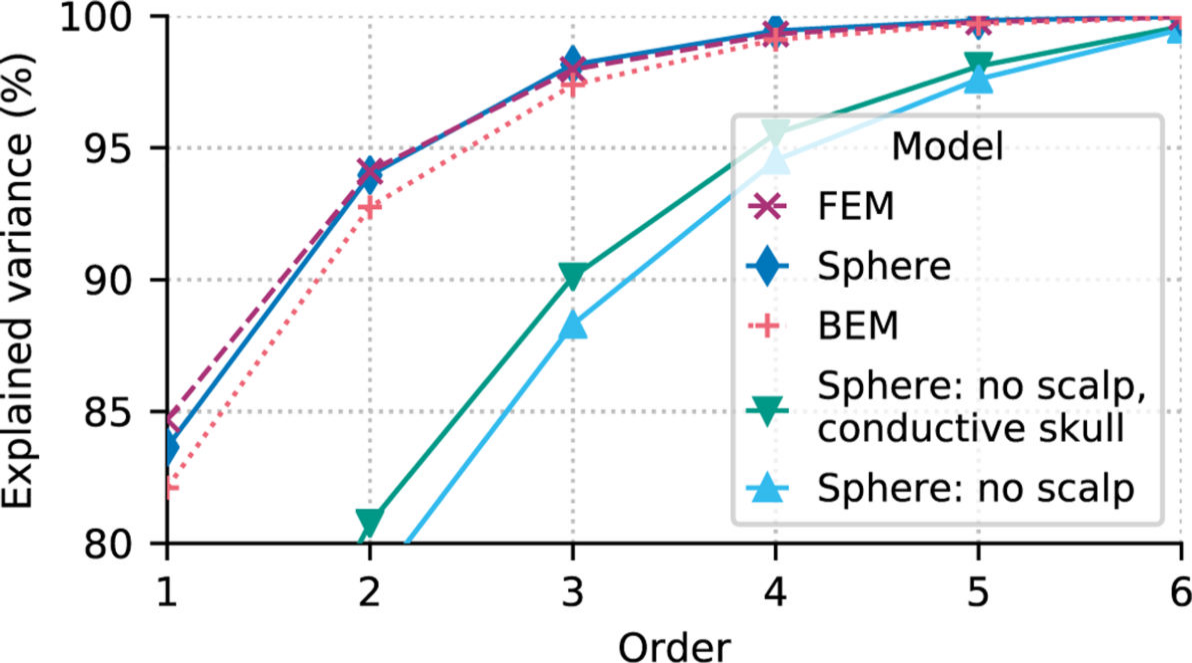
Explained variance convergence as a function of conductor model. Computing the variance explained in EEG signals for sources at 7 cm (free orientations) as a function of conductor model (individual lines) shows little difference between the convergence for the model type (spherical, BEM, or FEM). If the conductive skull and/or scalp were not present, higher orders would be required to explain the variance of the sources but the convergence towards 100% would still be fast.

Even for the most superficial sources, an order of *L*_EEG_ = 4 explains more than 98% of the signal variance, which indicates that the EEG signals are limited to less than ${\left(L_{\text{EEG}}+1\right)}^{2}=25$ degrees of freedom. This held true even when using a more accurate boundary element model to compute the forward solution using the MNE-Python default 3-layer BEM mesh (and conductivity values in S/M) consisting of the brain (0.3), skull (0.006), and scalp (0.3) with the linear collocation approach, or when the forward was computed using a 5-compartment finite element model (FEM) consisting of gray matter (0.33), white matter (0.14), CSF (1.79), skull (0.01), and scalp (0.43) computing using the SimBio pipeline [24] in FieldTrip [25]. Notably, when the skull or scalp were omitted from the spherical conductor model, higher expansion orders would be required to capture the forward model variance (Fig. 3). Nonetheless, even in those cases the saturation occurred at lower *l* (*L*_EEG_ = 6) than in the case of MEG (*L*_MEG_ = 8).

**Fig. 4.**
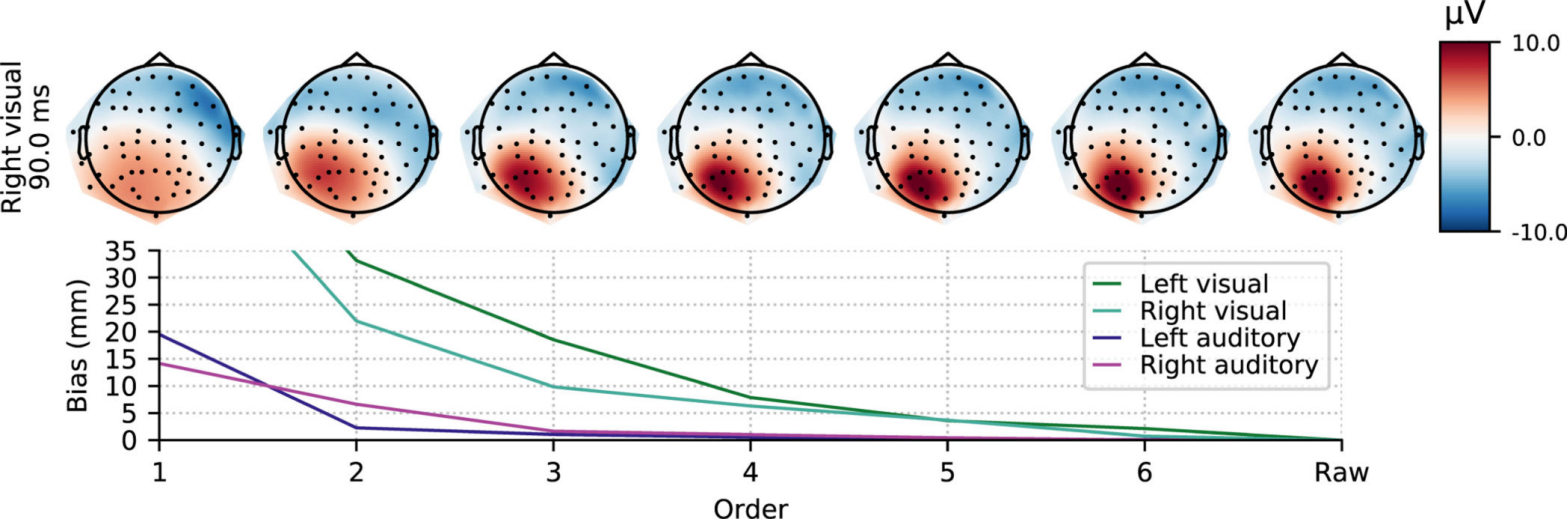
Bias of ECD fits induced by rank reduction. The ECD localization bias (y-axis; relative to the original data, rightmost point) for the four main conditions of the MNE-Python sample dataset (lines) falls below roughly 1 cm once an order of *l* = 4 (x-axis) or higher is is used. Topomap plots of the rank-reduced EEG data are shown for the right visual condition (top row) above the localization bias (bottom row). *l* = 1 order bias values that exceeded the plotting bounds were 73 and 62 mm for left and right visual conditions, respectively.

To look at an example of how expansion order relates to source localization, we took the trial-averaged data for the single adult subject in the MNE-Python sample dataset (for details, see [22]) under four conditions, each of which should evoke a clear response from a primary sensory region contralateral to the stimulation type: left auditory, right auditory, left visual, and right visual. We used the 3-layer BEM and head-to-MRI transformation supplied with that dataset to compute an equivalent dipole current (ECD) fit at 93 ms in each of these four conditions, as this was the time point where all conditions tended to have a clear peak. After computing ECD fits for the original data, we then projected the EEG data to a rank-reduced space using electrostatic moments with different expansion orders (see (53) below), computed ECD fits, and quantified localization bias as the distance between the rank-reduced ECD location and the ECD from the original data. As shown in Fig. 4, localization bias fell below around 1 cm once order *l* = 4 or higher was used.

Our experiments suggest that the MEG signal (or the corresponding electric field measurement), when considering a typical sensor arrangement based on SQUID technology, generally contains about 80 degrees of freedom while the EEG signals usually contain less than 40 degrees of freedom. It may seem surprising that the measurement of the electric potential contains significantly less spatial information about the underlying source than a measurement of the corresponding electric or magnetic field, even when reducing the smoothing effects of the conductor model. However, even without considering any effects caused by conductivity boundaries, the limited spatial information in the measured EEG signal can be understood by the fact that the electric potential is always a proportional quantity between two points. Mathematically, the potential difference between points A and B is the negative of the line integral of E(r) between these points:

(42) $$V_{A\to B}(t)=-\int_{A}^{B}E(r,t)\cdot \mathrm{dl},$$

which, according to (19), takes the form

(43) $$V_{A\to B}(t)=-c_{\text{e}}\sum_{l=0}^{\infty }\sum_{m=-l}^{l}a_{lm}^{e}(t)\int_{A}^{B}\frac{\nu_{lm}(\text{Ω})}{r^{l+2}}\cdot \text{dl}$$

for signals produced by the brain. Here, dl is an infinitesimal segment of the integration path. The term

$$\int_{A}^{B}\frac{\nu_{lm}(\text{Ω})}{r^{l+2}}\cdot \text{dl}$$

can be interpreted as a quantity proportional to the average of the VSH function of the order {*lm*} weighted by the radial term over the length of the path from A to B, which could attenuate spatially complex features, corresponding to high orders of *l*, along the path. Of course, it is in principle possible to arrange the EEG electrodes in such a way that a distribution of estimated values of the electric field could be obtained due to the relation E(r)=−∇V(r). Let us know briefly examine this in the Cartesian coordinates:

(44) $$E(r)=-\nabla V(r)=-\left(\frac{\partial V(r)}{\partial x}e_{x}+\frac{\partial V(r)}{\partial y}e_{y}+\frac{\partial V(r)}{\partial z}e_{z}\right),$$

where **e**_*x*_, **e**_*y*_, and **e**_*z*_ are the unit vectors pointing in the x-, y-, and z-directions. Without loss of generality, let us assume that we picked a pair of electrodes that are both located on the x-axis, i.e., $r_{1}={\left[\begin{array}{c} \begin{array}{ccc} x_{1} & 0 & 0 \end{array} \end{array}\right]}^{T}$ and $r_{2}={\left[\begin{array}{c} \begin{array}{ccc} x_{2} & 0 & 0 \end{array} \end{array}\right]}^{T}$. Thus, in the vicinity of these electrodes we can approximate the x-derivative as

(45) $$\frac{\partial V}{\partial x}\approx \frac{V_{2}-V_{1}}{x_{2}-x_{1}},$$

where $V_{j}\equiv V\left(r_{j}\right)-V\left(r_{\text{ref}}\right)$, j={1,2}, and **r**_ref_ corresponds to the location of a common reference electrode. Now $V_{2}-V_{1}=V\left(r_{2}\right)-V\left(r_{1}\right)$ and according to (42)

(46) $$V_{2}-V_{1}=-\int_{x_{1}}^{x_{2}}E_{x}(r)dx,$$

Because **dl** = **e**_*x*_*dx* in this case. Thus, based on the measurement of *V* (**r**), our estimate of the component *E*_*x*_ reduces to its mean value over the interval from *x*_1_ to *x*_2_:

(47) $${\hat{E}}_{x}=-\frac{V_{2}-V_{1}}{x_{2}-x_{1}}=\frac{\int_{x_{1}}^{x_{2}}E_{x}(r)dx}{x_{2}-x_{1}}.$$

Obviously, the shorter the distance $|\text{Δ}x|=\left|x_{2}-x_{1}\right|$ between the adjacent electrodes, the more accurate the estimate of *E*_*x*_ is. Note that for simplicity we dropped the time variable from (44)–(47) as the consideration was purely spatial.

Our theory and simulation results indicate that a measurement of the brain-induced electric *field* could yield more informative data than the present measurement of the corresponding on-scalp *potential* distribution, unless high-density EEG arrays can be applied that contain similar information as **E**(**r**) in terms of (44). However, to our knowledge measurements of brain-related electric field have never been conducted but there are some earlier simulation studies supporting the interpretation of our mathematical theory. Specifically, Petrov and Sridhar [26] demonstrated more focal neural source reconstruction when inverse modeling was based on the measurement of the electric field instead of the potential distribution.

### B. Signals of Interest and Interference in MEG and EEG

In MEG and EEG in particular, the space can be divided into an enclosed volume containing the object of interest and an open volume outside of it that extends to infinity (i.e., inside and outside the hypothetical spherical boundary in Fig. 1). Therefore, by setting the origin in a suitable location inside the brain volume, one only needs expansions of (19 and (20) to express the brain-related fields. We can, however, also add the expansion converging at the origin to describe the contribution of sources that are located in the volume $r^{\prime }>r$. Obviously, fields produced by such sources are not originating in the brain and can therefore be considered interference. We can now construct a separate signal subspace for the external contributions by substituting the terms in (34) and (41) by their counterparts in the expansions converging at the origin, see (17), and by calling the resulting subspaces **S**_in_ and **S**_out_, respectively. Then, for the multi-channel measurements of the magnetic and electric fields we have

(48) $$\text{d}(t)=\left[S_{\text{in}}S_{\text{out}}\right]x(t),$$

where the coefficient vector **x**(*t*) consists of the corresponding $a_{lm}^{\text{e}}(t)$ and $b_{lm}^{\text{e}}(t)$ or $a_{lm}^{\text{b}}(t)$ and $b_{lm}^{\text{b}}(t)$ coefficients, depending on whether we are measuring the electric or the magnetic field, respectively. Provided that the internal and external truncation orders can be kept low enough for the dimension of **S** = [**S**_in_ **S**_out_] to be smaller than the number of channels and the angle between subspaces **S**_in_ and **S**_out_ is large enough to guarantee a favorable condition number of **S**, we can decompose the data uniquely to the components

(49) $$\hat{x}(t)=S^{†}d(t),$$

where † indicates pseudoinverse. We can then suppress interference simply by reconstructing the data from the reconstructed internal multipole moments only:

(50) $${\hat{d}}_{\text{in}}(t)=S_{\text{in}}{\hat{x}}_{\text{in}}(t),$$

where ${\hat{\text{x}}}_{\text{in}}$ corresponds to the first *n* components of the vector ${\hat{\text{x}}}_{\text{in}}$. Note that for simplicity, we are not taking the effects of random sensor noise into account here. For details on such effects, see [15]. In the case of MEG, this approach has been termed signal space separation (SSS), and it has been successfully used with modern multichannel MEG systems for the purpose of suppression of external interference signals, standardization of signal representation by the multipole moments, as well as compensation for field distortions caused by head movements with respect to the rigid sensor array [15],[27]. Successful application of SSS has been demonstrated at least with systems consisting of a combination of magnetometer and planar gradiometer pickup loops [28] as well as systems containing axial gradiometer pickup loops [29], and in principle should be applicable to any multichannel MEG system.

**Fig. 5.**
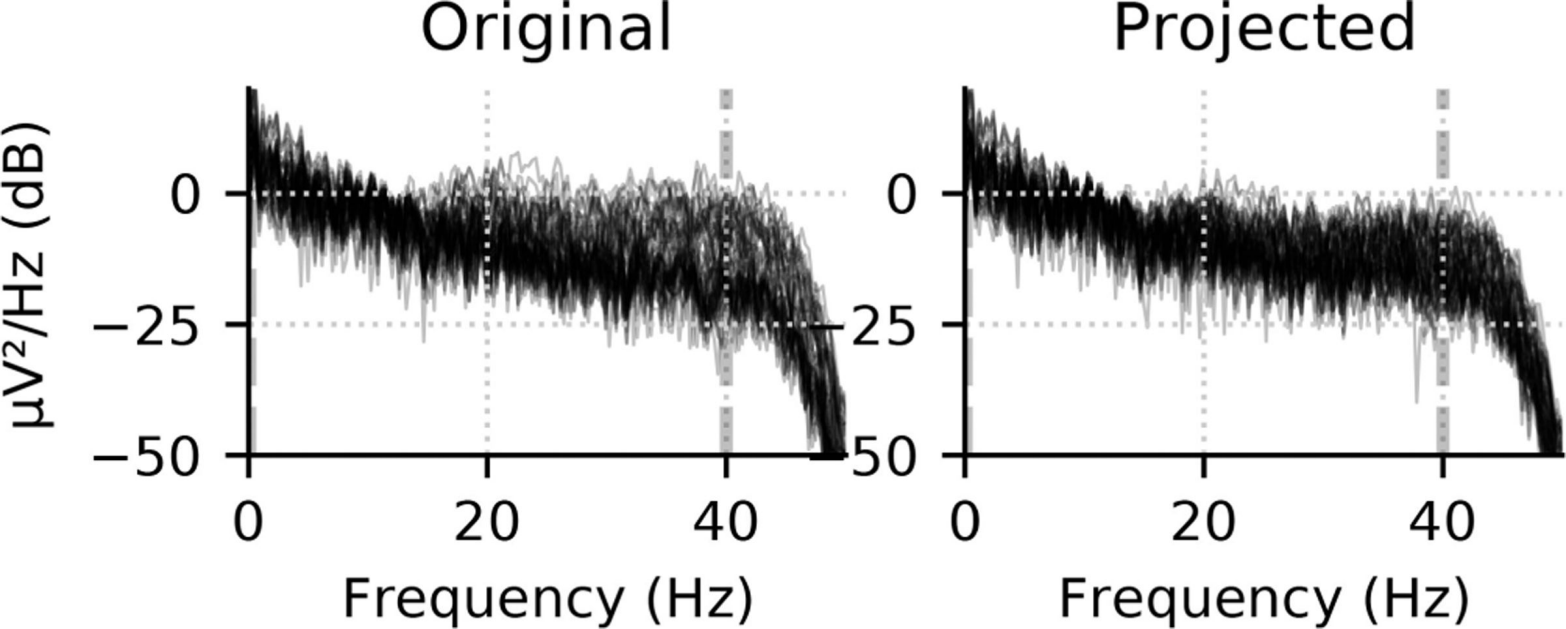
Reconstructing data from a low-dimensional subspace reduces noise. For the first 10 seconds of the MNE sample dataset, the power spectral density (original data left) shows a reduction in amplitude when projected to a low-dimension subspace (order *L*_in_ = 4) and back to the sensors (right).

The SSS basis vectors have been shown [15] to be linearly independent for any MEG sensor array that breaks the spherical measurement symmetry by having sensors that do not all point in the radial or in the tangential direction. This principle was demonstrated by boosting the separability of internal and external signals by adding a relatively small number of sensors that were tilted orthogonally to their neighboring sensors [30]. Another sufficient condition for breaking the spherical symmetry and achieving linear independence of the SSS basis is that not all sensors are located at the same distance from the origin. Since the magnetic field and the electric field are composed of the same basis functions, measurement of the brain’s electric or magnetic field will have the same conditions with respect to SSS, but traditional EEG measurements are more problematic in this sense because the electric potential is a scalar field, which means that the orientation of the electrodes does not provide any additional information. Therefore, we expect the SSS basis [**S**_in_ **S**_out_] calculated for EEG to be ill-conditioned (see Appendix). However, due to the low dimensionality of the EEG signals, one can apply a low-order basis **S**_in_ in a decomposition

(51) $${\hat{d}}_{\text{in}}(t)=S_{\text{in}}S_{\text{in}}^{†}d(t),$$

which will reconstruct the brain signals and attenuate any interference that has a relatively large subspace angle with **S**_in_. For example, the first 10 seconds of the MNE sample dataset [22], [23] in Fig. 5 shows a reduction in overall amplitude in the power spectral density estimates when data are reconstructed from these estimates, suggesting a reduction in the overall sensor noise levels. As for the suppression of external interference, it is possible that the temporally extended SSS approach (tSSS) [31] could be useful because it can be implemented as a temporal-domain interference detection algorithm even without explicitly modelling the spatial interference basis **S**_out_. See [32] for a related algorithm.

It should also be noted that MEG and EEG are not the only measurement modalities with geometry that allows us to divide the space into distinct “interesting” and “interfering” volumes. Another example could be, for example, geomagnetism and Gauss developed a similar mathematical model already in the early 19th century for the interpretation of data received from observatories located around the Earth [33].

### C. Standardization of Signal Distributions and Interpolation of Bad Channels

The signal dimensionality analysis presented in Section III-A indicates that the MEG and EEG signals are overdetermined, i.e., the number of measurements exceeds the underlying number of degrees of freedom, which is determined by the highest detectable *l*-components. It has already been shown in the case of MEG [15], [27] that an immediate application of this is that sensor-level signal distributions can be uniquely reconstructed from the measured data, even if the reconstructed MEG channels are not part of the original sensor array. In other words, one can use the VSH model for signal standardization and interpolation. Standardization can be defined as estimating the $a_{lm}^{\text{e}}$ or $a_{lm}^{\text{b}}$ components from the data and treating them as our signal, which is a device-independent representation of the recorded data. Again, the idea of signal standardization has already been described in MEG [15] and the same methodology immediately applies to the measurement of the electric field as well. In what follows, we describe the same idea for traditional EEG measurements with the modification compared to previous MEG work being the absence of **S**_out_ due to the aforementioned instability problems.

First, we define signal standardization in EEG as the operation

(52) $${\hat{x}}_{\text{in}}(t)=S_{\text{in}}^{†}\text{d}(t).$$

Now, assume that a subset of the EEG electrodes have been left out, e.g., due to excessive noise or other signal quality issues. Let us denote the updated signal subspace only containing the well-functioning electrodes as $S_{\text{in}}^{\prime }$. According to our dimensionality analysis in Section III-A, EEG signals are low dimensional and in data containing, e.g., 60 EEG channels, one could leave more than half of the channels out and still fully represent the detectable data by the measurement. The missing channels can be interpolated by first estimating the multipole moments based on the available basis and then reconstructing the signals with the signal basis of the desired EEG array as follows:

(53) $${\hat{x}}_{\text{in}}^{\prime }(t)=S_{\text{in}}^{\prime †}d(t),$$

(54) $$\hat{d}(t)=S_{\text{in}}{\hat{x}}_{\text{in}}^{\prime }(t).$$

**Fig. 6.**
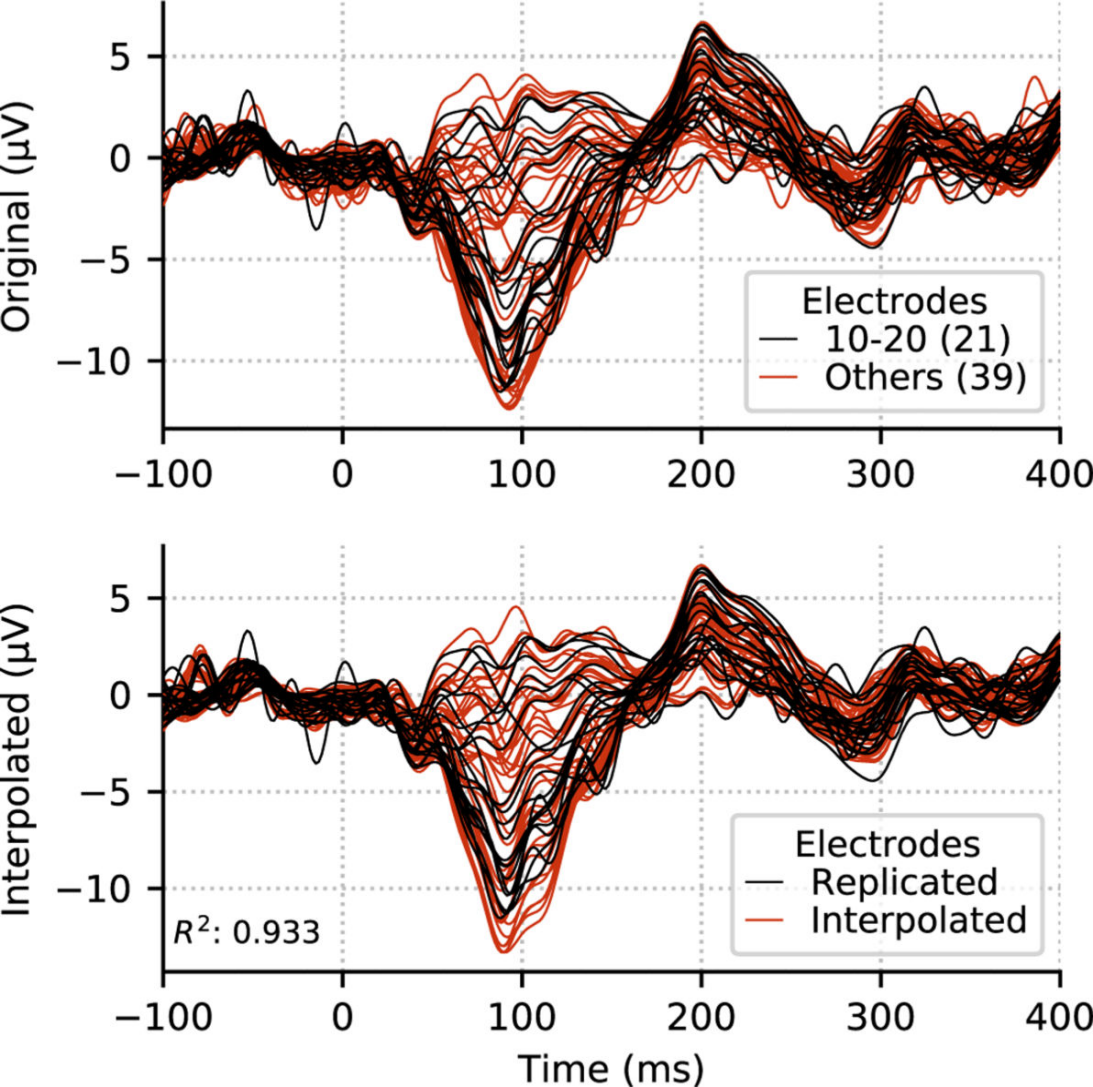
Reconstructing data using a subset of electrodes. When the 21 channels corresponding to a 10–20 layout (top, black) were used to interpolate the data to 39 other electrode locations (bottom, red) using the VSH expansion with order *L*_in_ = 2, the variance explained of the original data (top, red) by the and reconstructed data was over 93%.

To demonstrate the performance of this method, we analyzed an auditory evoked response recorded by a 60-channel EEG system (MNE sample dataset, auditory-left trials). We first removed 39 electrodes from the data, leaving 21 electrodes remaining that corresponded to the subset of the original 60 that fell within the standard 10–20 EEG electrode channel montage. When we reconstructed activity for the removed electrodes based on the data of the kept electrodes using just up to order *L*_in_ = 2, we observed good correlation between the actual and interpolated data, with more than 93% of the data variance explained (Fig. 6). The result demonstrates that in this case the standard 10–20 EEG electrode montage would have yielded essentially the same amount of information as the 60 channel layout. The good correlation between the actual and the interpolated signals also indicates that our approach provides one with an efficient interpolation method. One should note, however, that the 10–20 EEG electrode layout has a wide coverage over the head and the results would not be as good if the interpolation was based on, e.g., 21 electrodes only covering a small and restricted area above the brain. In addition, 21 electrodes would not provide enough information for spatially more complex EEG signals, such as the ones associated with visual responses.

### D. Calibration

**Fig. 7.**
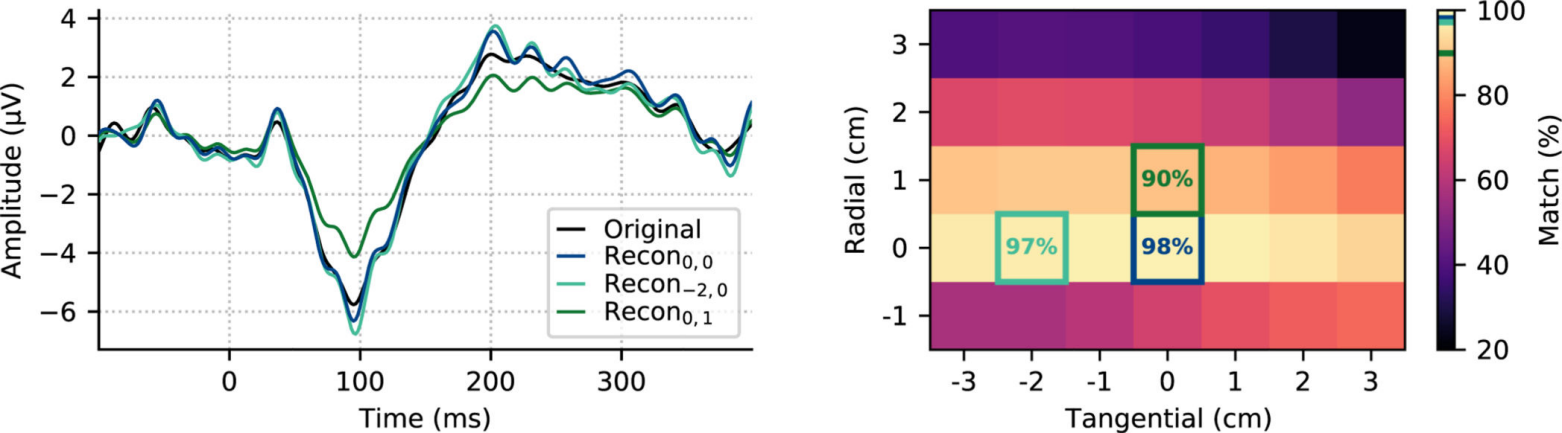
Signal reconstruction can be used to detect deviations from true locations. The original signal from a single sensor (left: black) was reconstructed from the other *N* − 1 channels using the VSH basis constructed using the original digitized electrode location (dark blue) or that location shifted in the radial direction (1 cm in green) or tangential (−2 cm in teal) direction. The peak of the variance of the original signal explained by the reconstruction (right) occurs at the true value, indicating that in principle VSH reconstructions could be used to detect deviations from correct digitized location.

In EEG, provided that truncation errors are negligible, there are two reasons for a significant mismatch between the basis **S**_in_ and recorded data **D**:

1. Errors in the electrode locations resulting in a distorted **S**_in_ matrix.
2. Contribution of external interference not explained by **S**_in_. However, as shown in Appendix, the subspace angle between **S**_in_ and **S**_out_ is very small in EEG, which indicates that calibration errors should be the primary source of model distortion. Fig. 7 is a demonstration of the effect of a simulated calibration error in case of a real EEG recording. We first estimated the electrostatic multipole moments up to order *L*_in_ = 4 according to (49) with **S** = **S**_in_, and then we moved the location of one of the electrodes in radial and tangential directions before reconstructing the sensor-level signals by (50). Our model explains 98% of the signal variance when the electrode is in the original digitized location. Introducing errors up to 1–2 cm in the tangential direction corresponded to relatively small changes in the explained variance, whereas equivalent radial shifts caused larger errors in the reconstructed signal.

Calibration of the EEG electrode array could be achieved by estimating the electrostatic multipole moments based on all electrodes and adjusting individual electrode locations to maximize the explained signal variance, after which the multipole moments are estimated again, followed by adjustment of the sensor positions as before. This procedure can be repeated in an iterative manner until the explained signal variance converges to some value.

## IV. Discussion

In this article we have presented the quasi-static electromagnetic fields as a combination of vector spherical harmonic functions whose amplitude coefficients (multipole moments) determine whether the basis functions describe the electric or magnetic counterpart of the EM field. Our main focus is on the modeling and measurement of the EM fields produced by the brain, but rather than deriving the formalism for the common modality pair MEG and EEG, we investigate MEG and the associated electric field, as opposed to the electric potential that EEG measures. This approach provides us with a symmetric mathematical formalism for the magnetic and electric interactions from which EEG’s properties can be easily derived assuming that the EEG signal can be modeled accurately enough with Laplace’s equation. Our formulae describe the relationship between current distributions giving rise to quasistatic EM fields in a manner that has no specific assumptions regarding the distribution of electric conductivity or spatial profile of the currents. Previous work on the unification of the magnetic and electric interactions in neuroimaging either investigates mathematical properties of the source space in detail [34], or is based on neuroscientific case studies [35]. As we let the source of the EM fields be any total current or charge concentration inside the object of interest, in our case the human head, the corresponding formalism on the EM fields is greatly simplified. Our formalism can be used as a concise starting point for more detailed analysis in terms of signal processing or analysis.

Due to the strict hierarchical organization of the VSH functions in terms of their amplitude decay as a function of measurement distance, our formalism can be used to study the essential number of spatial degrees of freedom contained in an EM field. As the electric and magnetic field components are composed of the same VSH functions, they have similar convergence properties as a function of distance, indicating that their dimensionality and information contents are also similar as the first approximation. On the other hand, the electric potential is proportional to a line integral of the electric field between two points, making it a scalar-valued field that is a spatially averaged outcome of a projection of the vector-valued electric field. This could smooth out some of the spatial features of the electric field, which decreases the effective dimensionality of the measurement. Although propagation through different head tissues is typically cited as the critical source of signal smoothing that reduces the resolution of EEG compared to MEG, our mathematical derivations suggest that the effective computation of a line integral over the electric field already acts as an important source of smoothing regardless of the conductivity profile of the head. In practice, EEG is the dominating measurement of the electric properties produced by the brain, but these results indicate that spatially more informative data could possibly be obtained by measuring the vector-valued electric field rather than the scalar electric potential. Previous simulation studies are in agreement with this conclusion [26]. One can estimate the electric field from the electric potential measurement by applying a spatial derivative operation to the EEG signal [36], but further studies are needed to investigate whether this approach yields similar information as the actual measurement of the electric field with distinct sensors with varying locations and orientations.

The feasibility of directly measuring the electric fields generated by neural sources remains to be explored. There have been recent advancements in the detection of weak electric fields but major challenges remain in the construction of such instruments. For example, SQUID-based electrometeters are sensitive to charges instead of the flux of the actual electric field. This is a complication as compared to the measurement of the magnetic flux through a loop (see, e.g., [37], [38]). In general, the main problem with the measurement of electric fields is the fact that the associated sensors tend to distort the electric field to be measured. These distortions are typically caused by surface charges or distortions due to the electrically conducting sensor material. A promising sensor type that does not cause significant field distortions is the microelectromechanical system (MEMS) device, which is operated by optically reading mechanical deformations of the sensor due to an external electric field [39]. Unfortunately, the sensitivity of the MEMS sensors is on the order of $100(\text{V}/\text{m})/\sqrt{\text{Hz}}$, which is not suitable for the neuroscience application.

Looking at the variance explained as a function of expansion order, we find that in EEG most of the data variance can be explained with a relatively low order expansion (e.g., *l* = 4, corresponding to 25 components). We also observe a similar trend for ECD localization bias in one example dataset. Consistent with existing work investigating the lower bounds for uncertainty for source localization in EEG [40] regardless of the density of sensors used [41], our results provide additional evidence that there may be an effective upper limit to the dimensionality of EEG, as higher expansion orders become of limited use (or detectability) once their amplitudes become too low relative to the sensor noise level. However, accounting for the structured nature and varying smoothness of the field patterns of brain sources as a function of source depth has been used to improve source localization accuracy (e.g., in multi-resolution inverse approaches like [42]). It remains an open question the extent to which these spherical harmonic expansions could inform inverse approaches, or provide a conclusive upper bound on the effective spatial resolution of EEG.

By applying the VSH model that provides us with a compact representation of the multi-channel data, we demonstrate linear algebra methods for decomposing the measured data into basic components that can be used for signal standardization across measurement geometries, channel interpolation, calibration, and suppression of external interference. For MEG, this is basically just a review of previously demonstrated methods [15], but by extending the VSH model to the electric field our simulations and experiment with a real data show that similar approaches can be applied in EEG as well. Moreover, if we were able to measure the electric field rather than potential, then MEG-specific methods such as SSS and tSSS would be readily available for the electric measurements as well.

In terms of source distribution, a particularly interesting outcome of the VSH methodology shown in this article is the fact that the forward models of the quasi-static magnetic field always reflect the tangential projection of the *total* electric current distribution only, with the tangential direction being relative to the chosen origin of the forward model. This result is independent of the geometry of the current distribution and conductor volume.

## V. Conclusion

In this article, we have reviewed the previously developed VSH theory for quasi-static magnetic fields and extended it to cover the full quasi-static electromagnetic field in a unified manner. The VSH model represents the continuous magnetic and electric vector fields as a set of hierarchically organized basis functions and their amplitude coefficients, which can be used as a standard representation of spatially sampled MEG and EEG measurements. The former measurement is a result of sampling the vector-valued magnetic field while the latter modality corresponds to sampling the electric potential, which is a scalar field. Our theory, however, unifies the mathematical representation of the electric and magnetic fields and we show that sampling of the electric field could have benefits over EEG and MEG in non-invasive brain imaging. The possible benefit over MEG can be accounted for the fact that the electric field is less restrictive to the direction of the underlying electric current. We also demonstrated practical applications in terms of signal processing and calibration that immediately follow from our theoretical framework.

## APPENDIX Linear Independence of the SSS Basis for EEG

According to (9) and (41), the EEG signal of channel *j*, taking both internal and external contributions (see Fig. 1) into account, is of the form

(55) $$v_{j}=c_{\text{e}}\sum_{l,m}\left(\frac{a_{lm}}{r_{j}^{l+1}}+b_{lm}r_{j}^{l}\right)Y_{lm}\left({\text{Ω}}_{j}\right).$$

Thus, for the SSS basis $\left[{\text{S}}_{\text{in}}\;{\text{S}}_{\text{out}}\right]$ calculated for EEG to be linearly independent, there should exist $a_{lm}\neq 0$ and $b_{lm}\neq 0$ such that

$$\left(\frac{a_{lm}}{r_{j}^{l+1}}+b_{lm}r_{j}^{l}\right)=0\;\forall j.$$

Obviously, one such solution is

(56) $$a_{lm}=-r_{j}^{2l+1}b_{lm}.$$

But since $a_{lm}$ and $b_{lm}$ do not depend on individual channels, (56) can only be valid if

(57) $$a_{lm}=-r_{0}^{2l+1}b_{lm},$$

where *r*_0_ is a constant value. In other words, for the SSS basis to be linearly dependent in the case of EEG, all electrodes would have to be on a spherical surface, i.e., *r*_*j*_ = *r*_0_ ∀*j*. Because the scalp is not exactly a spherical surface, one could conclude that SSS-based suppression of external interference is possible with EEG the same way as with MEG [15], but in practice the SSS basis of standard EEG arrangements is expected to have a very high condition number. This is due to the fact that the electrodes do not have the advantage of sampling different directions of a vector field (which has been found to be useful for MEG), and the scalp surface may not differ significantly enough from spherical symmetry to provide sufficient variation in the distances *r*_*j*_. Measurement of the electric field might provide an advantage for such applications.
